# Outcome of Direct Pulp Capping in Teeth Diagnosed as Irreversible Pulpitis: Systematic Review and Meta-Analysis

**DOI:** 10.4317/jced.59668

**Published:** 2022-07-01

**Authors:** Paula Ruiz-González, Daniel Cabanillas-Balsera, Juan J. Saúco-Márquez, Juan J. Segura-Egea

**Affiliations:** 1 DDS, Department of Stomatology, Section of Endodontics, School of Dentistry, University of Sevilla, C/ Avicena s/n, 41009-Sevilla, Spain; 2 DDS, MSc, PhD, Professor of Master in Clinical Endodontics, University of Sevilla, C/ Avicena s/n, 41009-Sevilla, Spain; 3 DDS, MD, PhD Professor of Master in Clinical Endodontics, University of Sevilla, C/ Avicena s/n, 41009-Sevilla, Spain; 4 MD, DDS, PhD, Professor, Department of Stomatology, Section of Endodontics, School of Dentistry, University of Sevilla, C/ Avicena s/n, 41009-Sevilla, Spain

## Abstract

**Background:**

This review and meta-analysis investigates the outcome of direct pulp capping in teeth diagnosed as irreversible pulpitis.

**Material and Methods:**

This systematic review includes experimental and descriptive clinical studies according to the PRISMA criteria, using PubMed and Scopus as database. We have included studies that performed direct pulp capping on human permanent teeth previously diagnosed with irreversible pulpitis and that carried out a subsequent follow-up. The outcome of interest was the clinical success of direct pulp capping.

**Results:**

A total of four studies met the inclusion criteria for this review, however only three of these could be included in the meta-analysis. These three studies represent a total sample of 62 teeth with irreversible pulpitis treated with direct pulp capping that showed an overall success rate of 0.953 (CI=0.900-1.005; *p*<0.001; I²=0). Additionally, the success rates of vital pulp therapies were compared, all of them being greater than 75%; and the success rates of the materials used were analyzed, giving values above 80% in all cases. The risk of bias of the included articles was established using the ROBINS-I tool, showing that two of the articles had a moderate risk of bias and the remaining two had a very high risk of bias.

**Conclusions:**

Based on the results of this review, direct pulp capping should be clinically included as a successful technique for the treatment of irreversible pulpitis. However, a larger number of studies with more rigorous methodologies are necessary to confirm the efficacy of this technique.

** Key words:**Irreversible pulpitis, direct pulp capping (DPC), vital pulp therapy (VPT), indirect pulp capping (IPC), partial pulpotomy, total pulpotomy.

## Introduction

Classically, the American Association of Endodontists has classified inflammatory pulp disease into two types, reversible pulpitis and irreversible pulpitis, the latter being the one that corresponds to the inability of the pulp organ to heal (1). In addition, irreversible pulpitis has been associated with persistent pain that does not disappear when the stimulus does, spontaneous pain and its increase at night and when the patient is placed in the supine position (2). The diagnosis of irreversible pulpitis correlated with the need for root canal treatment (RCT). On the contrary, the diagnosis of reversible pulpitis indicates vital pulp therapy procedures, such as indirect or direct pulp capping and partial or complete pulpotomy (3).

One of the vital pulp therapies for the treatment of teeth with reversible pulpitis is direct pulp capping (DPC), defined as the procedure in which a material (calcium hydroxide or an hydraulic calcium silicate cement), is directly placed over the exposed vital dental pulp to preserve dental vitality (4). DPC is considered as a minimally invasive approach in the treatment of deep caries lesions causing reversible pulpitis, avoiding the necessity to perform a root canal treatment (5). A recently published systematic review conclude a high success rate for DPC in teeth with cariously exposed pulps (6).

However, the results of studies carried out in the last years have shown that teeth diagnosed with irreversible pulpitis have achieved clinical cure after vital pulp therapy procedures, including DPC (13). These data suggest that considering RCT as the only therapy for teeth diagnosed with irreversible pulpitis could suppose an unnecessary overtreatment (7). Moreover, these results have led to propose a new classification of pulpal inflammatory pathology with much more conservative treatment options (9). On the other hand, maintaining vital dental pulp with DPC has several advantages: it preserves the structural integrity and the immunological functions of the tooth, it is a simpler technique that requires less time, less equipment and fewer materials from the dentist, and, in addition, it is a less expensive procedure for the patient, which causes less pain (9).

The objective of this systematic review and meta-analysis was to analyse the scientific literature investigating the outcome of DPC performed on teeth diagnosed with irreversible pulpitis.

## Material and Methods

-PICO question

The PICO question was as follows: in human permanent teeth diagnosed with irreversible pulpitis (P), is direct pulp capping (I), clinically successful (O)?

-Literature search strategy

Two examiners (PRG and JJSE) carried out the literature search in the PubMed and Scopus databases. The search included all articles registered up to December 23, 2021. For the search strategy, the following combination of MeSH terms and keywords was used: direct pulp capping AND (outcome OR success OR efficacy) AND pulpitis AND (irreversible OR symptomatic).

-Eligibility criteria

For the selection of articles, and in accordance with the PICO question, the following inclusion criteria have been taken into account: human clinical studies, including permanent teeth with preoperative diagnosis of irreversible pulpitis, treated with DPC, with assessment of the clinical outcome. Consequently, exclusion criteria were: case reports, experimental studies using extracted teeth, studies using non-human teeth or temporary teeth, clinical studies including teeth with reversible pulpitis or that did not make any classification of pulpal pathology, as well as those that performed other vital pulp therapy techniques or that they did not follow up on the case. No exclusions were made based on language or publication date of the study.

-Study selection

Two authors (PR-G and JJS-E) selected the studies. Firstly, duplicated studies were eliminated. In a second phase, those articles that, according to their title or the content of their abstract, failed to meet any of the inclusion criteria mentioned above were excluded. Finally, the remaining articles were read in full and those that, due to their content, did not meet the inclusion criteria or were systematic reviews were eliminated.

-Data extraction and quality assessment

Selected studies were assessed for internal risk of bias using the ROBINS-I tool (10). The following parameters were considered: random sequence generation (i), allocation concealment (ii), blinding of participants and personnel (iii), blinding of outcome assessment (iv), incomplete outcome data (v), selective notification (vi) and other biases (vii). The results were registered as present (+), absent (x) or unknown (-). To assess the risk of bias of each study, those that met only one or two parameters were considered to have a high risk of bias, those that met three or four parameters were classified as moderate risk of bias, and if they met five or six of the parameters were considered of low risk of bias.

-Meta-analysis

The primary outcome variable was the clinical success rate of DPC in teeth with irreversible pulpitis, expressed as a percentage, taking into account a 95% confidence interval.

The meta-analysis included those studies that took into account the preoperative pulp status and studied the clinical efficacy of DPC in them. Those studies that did not determine the pulp status of teeth that developed clinical failure after DPC treatment were excluded from the meta-analysis.

The OpenMetaAnalyst software, was used to perform the meta-analysis. As the included studies had different designs and the sample size was small, the random effects inverse variance model was used in the meta-analysis to determine the success rate. The meta-analysis was represented in a forest plot.

To estimate the heterogeneity of the included studies, the I² value was calculated. Heterogeneity was considered high when the I² value was greater than 75%, moderate if it was between 50% and 75%, slight if it was between 25% and 50%; and low if it was less than 25%.

## Results

-Literature search an study selection

**Figure 1:**
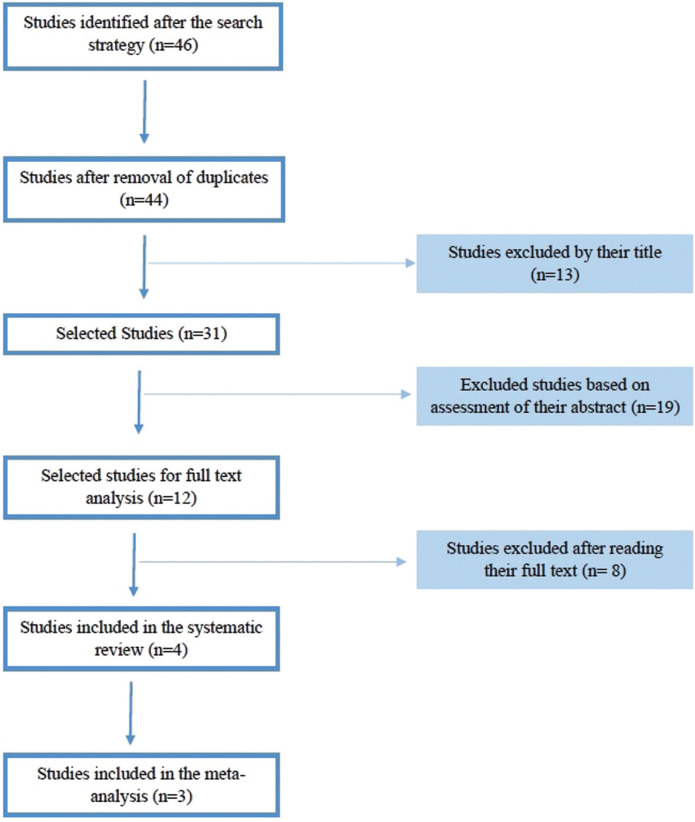
Flow diagram of the search strategy of the systematic review and meta-analysis.

After the literature search, 46 studies were selected, which after eliminating duplicates, made up 44 studies (Fig. 1). After the analysis of the titles, 13 studies were excluded. Of the remaining 31 studies, 19 were excluded after reviewing their abstracts. Finally, a detailed reading of the complete texts of the 12 selected studies was carried. After complete reading, eight articles did not met the inclusion criteria and were eliminated (Table 1). Therefore, four studies were included in the systematic review: Asgary *et al*. (2018) (11), Parinyaprom *et al*. (2018) (12), Asgary *et al*. (204) (13) and Matsuo *et al*. (1996) (16).

**Table 1:**
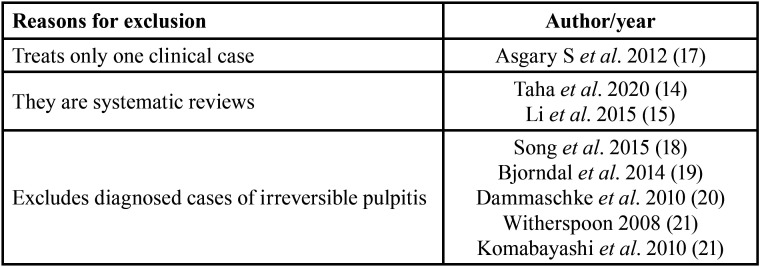
Excluded articles and reasons for the exclusion.

-Characteristics of included studies

The general characteristics of the four studies included in this review are summarized in Table 2. All of them deal with more or less extensive samples of teeth with deep caries, the removal of which most likely leads to exposure of the pulp tissue. The studies carried out by Asgary *et al*. in 2018 and 2014 (11,13), seek to compare the efficacy of the different Vital Pulp Therapies (IPC, DPC, partial pulpotomy and total pulpotomy) in permanent teeth with closed apex, that is, mature, with a diagnosis of irreversible pulpitis. In addition, the 2018 study (11) also studies the impact of suffering from apical periodontitis on the aforementioned teeth. On the other hand, the study by Paranyaprom *et al*. in 2018 (12) compares the DPC technique performed with ProRoot MTA or Biodentine in teeth with healthy pulp, reversible pulpitis or irreversible pulpitis. Finally, the study by Matsuo *et al*. (1996) (16) investigated the influence of various preoperative and intraoperative factors on the success of DPC procedure in teeth with pulp exposures due to caries in all cases.

**Table 2:**
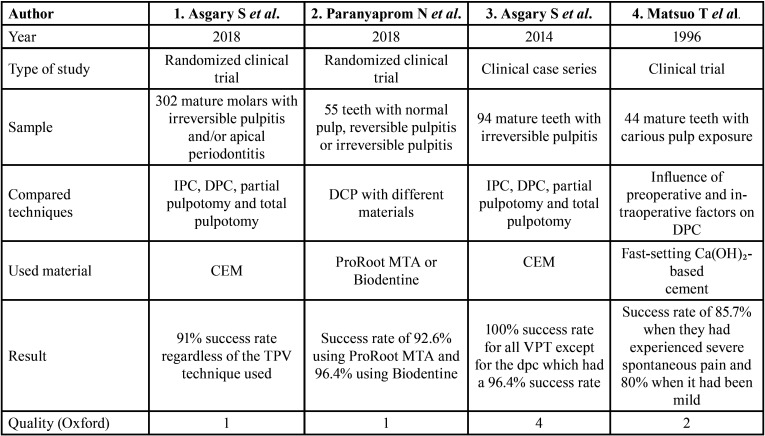
General characteristics of included studies.

Taking into account the samples of the different studies, these differ greatly between them. Asgary’s study in 2018 (11) presents a very large sample of 302 participants, 73 of whom were treated with DPC, 84 treated with IPC, 76 treated with partial pulpotomy, and 69 with total pulpotomy. In the Paranyaprom study in 2018 (12), the sample is much smaller, with 55 teeth involved, of which 27 were treated with ProRoot MTA and 28 with Biodentine. In the Asgary study in 2014 (13), the sample used was 94 teeth, 28 of them treated with IPC, 28 with DPC, 29 with partial pulpotomy and 9 with total pulpotomy. Finally, in the study by Matsuo in 1996, the sample had 44 teeth, of which only 11 had irreversible pulpitis, treating as a diagnostic factor that they had experienced spontaneous pain prior to treatment, whether severe or mild. The rest of the sample denied having previously experienced spontaneous pain.

Taking into account the procedure used for the DPC technique, it was very similar in all the articles. In the first three articles (11-13) local anesthesia was applied and absolute isolation was performed with a rubber dam, however, in the study by Matsuo (16) isolation was performed without the application of anesthesia and the removal of the carious lesion until the patient manifested pain, at which time it was decided to administer anesthesia. All used lidocaine with epinephrine (1:80,000) as anesthetic agent, except for the Paranyaprom study in 2018 (12) that used articaine with epinephrine (1:100,000). It should be noted that in the study by Matsuo (16) the anesthetic used is not specified. Subsequently, in all cases the caries was completely removed and the pulp tissue was exposed. Next, for the cleaning of the cavity and the production of hemostasis of the pulp, the Asgary study in 2018 (11) used saline as an irrigator and placed a sterile cotton ball soaked in 0.2% chlorhexidine for 5 minutes to achieve hemostasis. If this was not achieved, a sterile cotton ball soaked in 5.25% sodium hypochlorite was placed. However, in the rest of the articles, the agent used for this purpose is sodium hypochlorite with a concentration of 2.5% in the Paranyaprom study in 2018 (12), 5.25% in the Asgary study in 2014 (13) and 10% in the study by Matsuo in 1996 (16), which carried out successive washes with hypochlorite and hydrogen peroxide at 3%. Focusing on the DPC itself, two of the studies (11,13) used CEM as the material while the Paranyaprom study used ProRoot MTA or Biodentine and the Matsuo study used different brands of quick-setting calcium hydroxide-based cements. In Asgary’s study in 2018 (11), the CEM was covered with resin-modified glass ionomer (Vitrebond) and the sample teeth were filled with composite resin. On the other hand, in the Paranyaprom study (12), only glass ionomer was placed on MTA and not on Biodentine, and the teeth were restored with composite resin, amalgam or metal crowns depending on the amount of destruction they presented. In the study by Asgary in 2014 (13), the studied teeth were filled with composite resin, silver amalgam or ionomer and in the study by Matsuo in 1996 (16) calcium hydroxide-based cement was covered with zinc oxide-eugenol cement and temporarily restored for 3 months with glass ionomer. Subsequently, if the tooth showed no signs of failure, it was permanently filled.

Finally, the results of the included studies, show a success rate of the different treatment modalities analysed remarkably high, exceeding 90% in the majority of the cases. Specifically, in the study by Asgary in 2018 (11), the success rate is 91%, regardless of the VPT technique used and the presence or absence of periapical pathology. On the other hand, in the Paranyaprom study (12), the global success rate was 94.5%, being 92.6% in the treatments carried out with ProRoot MTA and 96.4% in those carried out with Biodentine, without statistical significant differences between the two. In Asgary’s study in 2014 (13), only one of the treated teeth failed and developed postoperative signs and symptoms of inflammation, which had been treated with DPC, thus the success rate for IPC, partial pulpotomy and total pulpotomy was 100%, reducing to 96.4% in the case of DPC. Lastly, in the study by Matsuo in 1996 (16) the success rate was lower than in previous studies, being 85.7% in cases where the patient had experienced severe spontaneous pain prior to treatment and this rate was reduced to 80% if the spontaneous pain experienced by the patient had been mild.

-Specific characteristics:

The specific characteristics of each of the articles are summarized in Table 3.

**Table 3:**
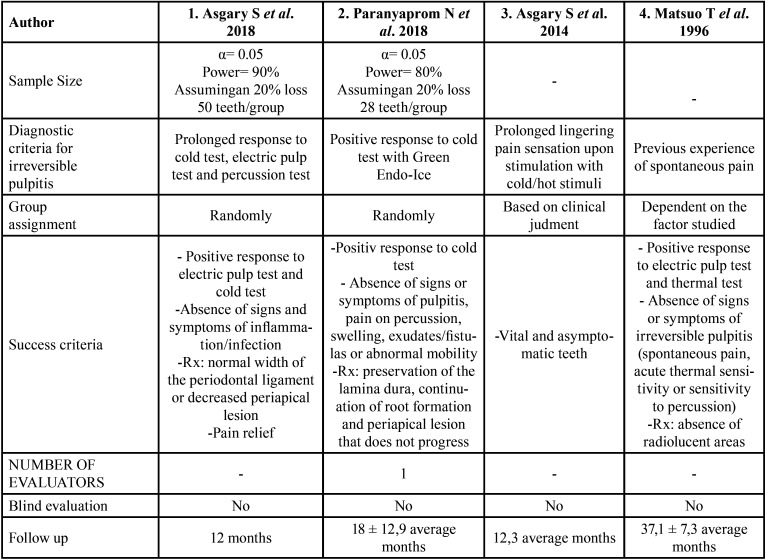
Specific characteristic of included studies.

Considering the samples of each of the studies, only the studies by Asgary in 2018 (11) and Paranyaprom in 2018 (12), specify how they have calculated the sample size. Both have determined an α= 0.05, however Asgary uses a power of 90% while Paranyaprom determines a value of 80% for the power. As both studies assume a loss of 20%, the sample size for the 2018 Asgary study (11) will be 50 cases for each treatment and in the Paranyaprom study (12) it will be 28 cases for each treatment. Regarding the assignment of the patients to the different groups, the first two studies (11,12) carried it out randomly. The study by Asgary *et al*. (2014) (13) selected each type of treatment to be carried out according to the clinical judgment of the operator, whereas in the study by Matsuo *et al*. (1996) (16), patients were classified into different groups according to the preoperative or intraoperative factor to be studied. In the case of evaluating the history of dental pain, which is the factor that concerns this review, patients are divided into 4 groups according to whether the spontaneous pain has been severe, mild, has not existed or is uncertain.

Regarding the operators, it should be noted that in the first article (11) they were dentists from the operative dentistry department of the Imam Khomeini Dental Clinic, Tehran, Iran. In the second (12), the operators were postgraduate students supervised by a professor, in the third article (13), the operator was a specialized endodontist, and in the fourth article (16) no data about the operators is specified.

Regarding the diagnostic criteria used to diagnose reversible pulpitis, Asgary *et al*. (2018) considered as a diagnostic criterion that the tooth has an increased response to the electrical test, the thermal test and percussion. However, Paranyaprom *et al*. (2018) considered sufficient that the tooth had a positive response to the Green Endo-Ice cold test. On the other hand, the criterion used by Asgary *et al*. (2014) was that the affected tooth experienced a sensation of prolonged and persistent pain after a thermal stimulus. Finally, Matsuo *et al*. (1996) considered the diagnosis of irreversible pulpitis when the carious tooth presented history of spontaneous pain.

Regarding the success criteria of the treatment carried out, each study selects a series of items that the treated teeth must meet. These are very similar for all the studies and are summarized in the absence of signs or symptoms of pulpal inflammation, the absence of signs of pulpal pathology in the radiographic tests, and the positive response in the different electrical or thermal tests.

On the other hand, none of the studies state that the evaluation of the cases is carried out blindly, in fact, only the article by Paranyaprom (12) specifies that an unblinded operator decided the final result for each tooth under consideration with the clinical and radiographic evaluation of the same.

Finally, it is worth noting the different follow-up periods followed by the selected studies. The 2018 Asgary study (11) has a follow-up of 12 months, the 2018 Paranyaprom study (12) has a follow-up of 18 ± 12.9 months, while the mean follow-up in the Asgary study of 2014 (13) is 12.3 months. On the other hand, the study by Matsuo from 1996 (16) is the one with the longest follow-up, with a mean of 37.1 ± 7.3 months.

-Additional analysis

•Comparison of the success of the different VPT

In view of the secondary objectives of this systematic review, the two articles written by Asgary in 2018 and 2014 (11)(13) have been selected to compare the success rates, expressed as percentages, of the different vital pulp therapy techniques: IPC, DPC, partial pulpotomy and total pulpotomy.

In the 2014 study, the success rates for both IPC and partial and total pulpotomy are 100%, falling to 96.4% in the case of DPC. However, in the 2018 study, the success rates are lower, specifically the IPC shows a success rate of 94.9%, the DPC of 86.5%, in partial pulpotomy it is 77.5% and in the total pulpotomy of 86.7%. It should be noted that the samples used in the 2018 Asgary study are more numerous for all the techniques than those used in the 2014 study. All these data are detailed in Table 4.

**Table 4:**
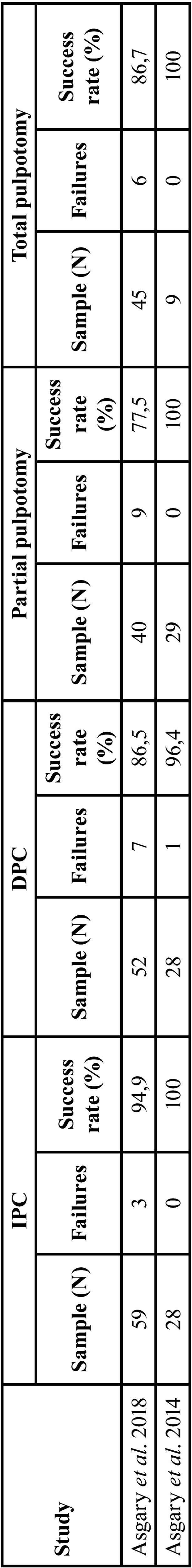
Comparison of the success of the different VPT.

•Comparison of the success of different materials used for RPD:

Another of the secondary objectives of this review is the comparison of the efficacy of the different materials used in the selected articles for the performance of DPC in teeth with irreversible pulpitis. To do this, the success rates shown by ProRoot MTA and Biodentine used in the 2018 Parinyaprom study (12) have been studied, as well as the efficacy expressed in the success rate of fast-setting calcium hydroxide-based cement used by Matsuo in his 1996 study (16).

As detailed in Table 5, ProRoot achieved a success rate of 92.6% in the Parinyaprom study, while Biodentine achieved a slightly higher rate, specifically 96.4%. On the other hand, the calcium hydroxide-based cement that Matsuo used in his study reported a lower success rate of 81.8%.

**Table 5:**
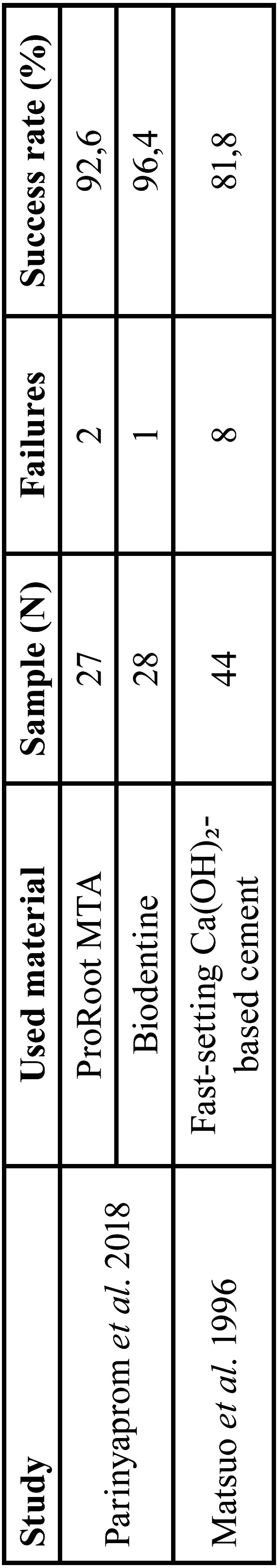
Comparison of the success of different materials used for DPC.

-Quality assessment

**Figure 2:**
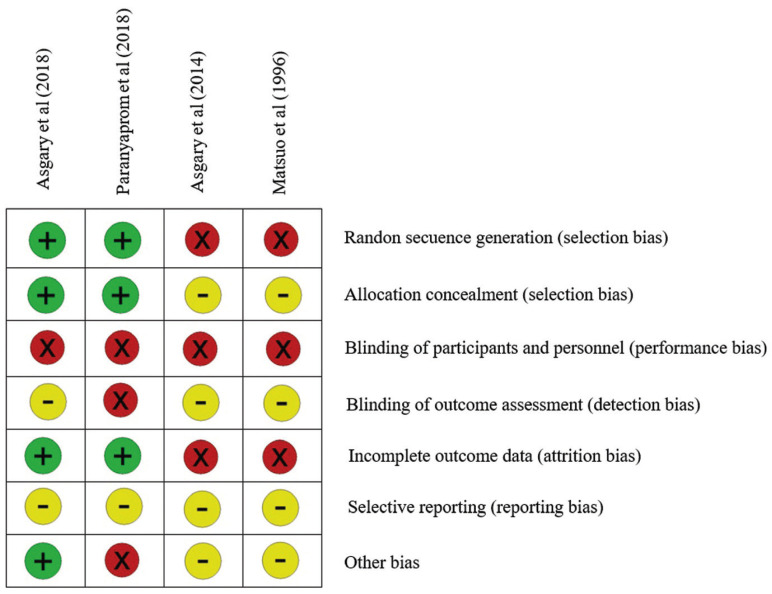
Quality assessment and risk of bias evaluated for each study.

When evaluating the methodological quality and the risk of bias of each of the articles included in the review (Fig. 2) following the parameters established by the ROBINS-I tool, it is found that both the study by Asgary (2018) (11) and the study by Paranyaprom (2018) (7) only meets three of the parameters mentioned above, therefore they would be studies with a moderate risk of bias. On the other hand, the study by Asgary (2014) (13) and the study by Matsuo (1996) (16) do not meet any of the parameters, three of them being unknown and three absent, therefore the risk of bias of these two items is sky high.

It should be noted that the only criteria that are met in the two 2018 studies (11)(12) are the random assignment of each patient to one of the study groups and the blind assignment for them, which indicates the absence selection bias. In addition, both have data on the cases of abandonment that have occurred in each study, so they do not carry out attrition bias.

-Meta-analysis

For the meta-analysis, the studies by Parinyaprom from 2018 (12), Asgary from 2014 (13) and Matsuo from 1996 (16) were included. Therefore, Asgary’s 2018 study (6) was excluded because it did not specify the preoperative pulpal status of DPC-treated teeth that had developed clinical failure, making it impossible to calculate the success rate of DPC in only teeth with previous diagnosis of irreversible pulpitis. Therefore, a total of 62 teeth with irreversible pulp status were studied for response to DPC treatment.

The heterogeneity between the studies was determined using the Tau² test, resulting in a value of 0.000 (Q = 1.342; *p*= 0.511). The value of I² was also 0, so it was concluded that the heterogeneity between the studies was low.

In addition, the general success rate of the meta-analysis was calculated, resulting in 0.953, with a confidence interval of 95% (CI= 0.900-1.005; *p*<0.001), which indicates that the use of DPC in teeth with irreversible pulpitis is a technique that causes healing of the pulp in a high percentage of cases, at least understood as the absence of clinical symptoms of pulp disease.

**Figure 3:**
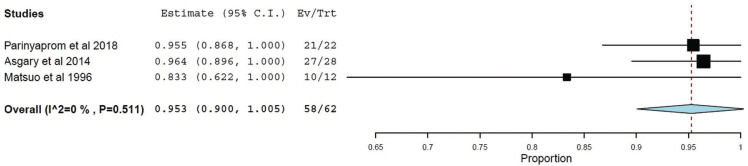
Forest plot of the included studies.

Figure 3 shows the results obtained in the three articles included in the meta-analysis represented by a forest plot.

## Discussion

In this review, the clinical efficacy of the application of the DPC technique in teeth that had previously been diagnosed with irreversible pulpitis has been studied. To this end, four studies dealing with mature human teeth with a correct pulpal diagnosis have been chosen and the success rate of the DPC after postoperative follow-up of the patient has been calculated for each of the samples. Thus, it has been concluded that RPD achieves very high success rates, in all cases above 80%, even though the preoperative pulp diagnosis was compromised.

Therefore, the result of this review shows that the application of DPC should be a therapeutic option to consider for the treatment of teeth that develop irreversible pulpitis, rejecting the idea that the only treatment for these teeth is the application of the traditional treatment of ducts. The result of this review should also be considered within the knowledge of minimally invasive dentistry, which is having such a boom in the world of dentistry in recent years.

It must be also highlight that this review has compared the clinical results of DPC with other VPT techniques, such as IPC, partial pulpotomy and total pulpotomy, obtaining as a result that the success rate of all of them in teeth with irreversible pulpitis is very similar, the differences not being statistically significant, so the rest of the VPT techniques should be taken into account as possible treatments in irreversible pulp diagnoses.

These results coincide with other reviews, such as that by Taha *et al*. (2020) (14), which show the importance of considering minimally invasive techniques for maintaining the vitality of the pulp; the study by Asgary *et al*. (2018) (11) that demonstrates the clinical success of VPT regardless of the technique used or the review by Aguilar *et al*. (2011) (23) that indicates the use of RPD in permanent teeth as a successful treatment with exposed pulp for carious reasons.

In addition, it should be taken into account that numerous studies (11,12) ensure that the clinical pulp status, which was classically defined with diagnostic tests such as the cold test or the history of spontaneous pain, also used by the studies analyzed in this review, are not correlated correctly with the pulpal histological status or with the negative progression of the disease in what has traditionally been called irreversible pulpitis. That is why the new Wolters classification of pulpal inflammatory disease of the hand (9) emerged, which should be used in subsequent research studies on the different treatment alternatives for inflammatory pulp disease.

Perhaps it is also important not to forget that the pulp is nothing more than an organ of the human body and therefore, like all other organs, it should reduce its inflammation by removing the infectious agent that produces it, as Mejare *et al*. (2012) (24) in his work. Therefore, with the simple removal of the carious process, the host’s immune system should eliminate all the inflammatory infiltrate of the pulp and regenerate completely (12).

On the other hand, it was studied whether the material used to perform DPC could influence its clinical success rate, observing that there are no statistically significant differences between the use of ProRoot MTA, Biodentine or Cement based on Ca(OH)2 of fast setting, although the latter presented a slightly lower success rate. It must be considered that all these materials are biocompatible, which is why they favor pulpal healing and produce a bacteria-free environment around the pulpal tissue due to their excellent sealing, therefore, as Farzaned *et al*. (2004) indicates, the quality of the restoration and the biocompatible materials used will promote a successful progression of the cases.

However, this review has had numerous limitations. Unfortunately, there is a very low volume of articles that study the efficacy of RPD in irreversible pulpitis, with only four meeting the inclusion criteria imposed, and of these, only three could be included in the meta-analysis. In addition, all the studies are of limited quality, with two of them (11,12) having a moderate risk of bias and the remaining two (13,16) having a very high risk of bias. It is surprising that none of the studies blinded the operators or the evaluators, even though two of them were randomized clinical trials and all four articles were published in high-impact scientific journals.

With regard to the Asgary study in 2018, we must point out that although it has previously been stated that the groups were assigned randomly, this is not entirely true, since those cases that did not present pulp exposure after caries removal were directly included in the group treated with IPC. Also in this study, it is very striking that the author did not specify the pulpal state that the teeth that failed treatment presented preoperatively, which meant that this study could not be included in the meta-analysis of this review.

Another limitation found was the low volume of cases presented by the rest of the included studies (12,13,16), being especially low in the study by Matsuo (1996). From this last study, the very peculiar methodology that it uses must be highlighted since it decides to start the operation of the pieces without the placement of anesthesia until the patient’s complaint is received, which is completely unthinkable today. This evidences the age of the study, which perhaps further diminishes its clinical relevance.

## Conclusions

The results of this review with meta-analysis could lead to the affirmation that DPC is a successful technique in the treatment of teeth diagnosed with irreversible pulpitis, but due to the scarce number of articles and the poor quality of the evidence, they are needed studies with larger samples and more rigorous evaluation methods.

## References

[1] Ali SG, Mulay S (2015). Pulpitis: A review. Journal of Dental and Medical Sciences.

[2] American Association of Endodontists (2009). AAE Consensus Conference Recommended Diagnostic Terminology. Journal of Endodontics.

[3] American Association of Endodontists (2013). Endodontic Diagnosis. Endodontics: Colleagues for Excellence.

[4] Hilton TJ, Ferracane JL, Mancl L, NWP) for NPRC in ED (2013). Comparison of CaOH with MTA for Direct Pulp Capping: A PBRN Randomized Clinical Trial. Journal of Dental Research.

[5] Giacaman RA, Muñoz-Sandoval C, Neuhaus KW, Fontana M, Chałas R (2018). Evidence-based strategies for the minimally invasive treatment of carious lesions: Review of the literature. Advances in clinical and experimental medicine : official organ Wroclaw Medical University.

[6] Cushley S, Duncan HF, Lappin MJ, Chua P, Elamin AD, Clarke M (2021). Efficacy of direct pulp capping for management of cariously exposed pulps in permanent teeth: a systematic review and meta-analysis. International Endodontic Journal.

[7] Crespo-Gallardo I, Hay-Levytska O, Martín-González J, Jiménez-Sánchez MC, Sánchez-Domínguez B, Segura-Egea JJ (2019). Correction: Criteria and treatment decisions in the management of deep caries lesions: Is there endodontic overtreatment?. Journal of clinical and experimental dentistry.

[8] Careddu R, Duncan HF (2021). A prospective clinicla study investigating tbe effectiveness os partial pulpotomy after relating preoperative symptons to a new and established classification od pulpitis. Internarional Endodontic Journal.

[9] Wolters WJ, Duncan HF, Tomson PL, Karin IE, McKenna G, Dorri M (2017). Minimally invasive endodontics: a new diagnostic system for assessing pulpitis and subsequent treatment needs. International Endodontic Journal.

[10] Alarcón Palacios M, Ojeda Gómez RC, Ticse Huaricancha IL, Cajachagua Hilario K (2015). Critical analysis of randomized clinical trials: The risk of bias. Rev Estomatology Herediana.

[11] Asgary S, Hassanizadeh R, Torabzadeh H, Eghbal MJ (2018). Treatment Outcomes of 4 Vital Pulp Therapies in Mature Molars. J Endod.

[12] Parinyaprom N, Nirunsittirat A, Chuveera P, Na Lampang S, Srisuwan T, Sastraruji T (2018). Outcomes of Direct Pulp Capping by Using Either ProRoot Mineral Trioxide Aggregate or Biodentine in Permanent Teeth with Carious Pulp Exposure in 6- to 18-Year-Old Patients: A Randomized Controlled Trial. J Endod.

[13] Asgary S, Fazlyab M, Sabbagh S, Eghbal MJ (2014). Outcomes of different vital pulp therapy techniques on symptomatic permanent teeth: a case series. Iran Endod J.

[14] Taha NA, About I, Sedgley CM, Messer HH (2020). Conservative Management of Mature Permanent Teeth with Carious Pulp Exposure. J Endod.

[15] Li Z, Cao L, Fan M, Xu Q (2015). Direct Pulp Capping with Calcium Hydroxide or Mineral Trioxide Aggregate: A Meta-analysis. J Endod.

[16] Matsuo T, Nakanishi T, Shimizu H, Ebisu S (1996). A clinical study of direct pulp capping applied to cariousexposed pulps. J Endod.

[17] Asgary S, Nosrat A, Homayounfar N (2012). Periapical healing after direct pulp capping with calcium-enriched mixture cement: a case report. Oper Dent.

[18] Song M, Kang M, Kim HC, Kim E (2015). A randomized controlled study of the use of ProRoot mineral trioxide aggregate and Endocem as direct pulp capping materials. J Endod.

[19] Bjørndal L, Demant S, Dabelsteen S (2014). Depth and activity of carious lesions as indicators for the regenerative potential of dental pulp after intervention. J Endod.

[20] Dammaschke T, Leidinger J, Schäfer E (2010). Long-term evaluation of direct pulp capping--treatment outcomes over an average period of 6.1 years. Clin Oral Investig.

[21] Witherspoon DE (2008). Vital pulp therapy with new materials: new directions and treatment perspectives--permanent teeth. Pediatr Dent.

[22] Komabayashi T, Zhu Q (2010). Innovative endodontic therapy for anti-inflammatory direct pulp capping of permanent teeth with a mature apex. Oral Surg Oral Med Oral Pathol Oral Radiol Endod.

[23] Aguilar P, Linsuwanont P (2011). Vital pulp therapy in vital permanent teeth with cariously exposed pulp: a systematic review. J Endod.

[24] Mejare I, Axelsson S, Davidson T, Frisk F, Hakeberg M, Kvist T (2012). Diagnosis of the condition of the dental pulp: a systematic review. Int Endod J.

